# Influences on Physical Activity Participation Among Older Adults: Perspectives of Exercise Professionals and Older Adult Exercise Participants

**DOI:** 10.3390/ijerph22030371

**Published:** 2025-03-03

**Authors:** Heather M. Hanson, Alia Bharwani, R. Stewart Longman, Marc J. Poulin

**Affiliations:** 1 Department of Community Health Sciences, Cumming School of Medicine, University of Calgary, 3D10, 3280 Hospital Drive NW, Calgary, AB T2N 4Z6, Canada; 2 Provincial Seniors Health and Continuing Care, Alberta Health Services, 10301 Southport Lane SW, Calgary, AB T2W 1S7, Canada; 3 Faculty of Kinesiology, University of Calgary, 2500 University Drive NW, Calgary, AB T2N 1N4, Canadapoulin@ucalgary.ca (M.J.P.); 4 Hotchkiss Brain Institute, Cumming School of Medicine, University of Calgary, 3330 Hospital Drive NW, Calgary, AB T2N 4N1, Canada; stewart.longman@albertahealthservices.ca; 5 Psychology Services, Foothills Medical Centre, Alberta Health Services, 1403 29th Street NW, Calgary, AB T2N 2T9, Canada; 6 O’Brien Institute for Public Health, Cumming School of Medicine, University of Calgary, 3280 Hospital Drive NW, Calgary, AB T2N 4Z6, Canada; 7 Department of Physiology & Pharmacology, Cumming School of Medicine, University of Calgary, 3330 Hospital Drive NW, Calgary, AB T2N 4N1, Canada; 8 Libin Cardiovascular Institute of Alberta, Cumming School of Medicine, University of Calgary, HMRB, 3310 Hospital Drive NW, Calgary, AB T2N 4N1, Canada; 9 Department of Clinical Neurosciences, University of Calgary, Room 1195, FMC, 1038 29th Street NW, Calgary, AB T2N 2T9, Canada; 10 Brenda Strafford Foundation Chair in Alzheimer Research, Cumming School of Medicine, University of Calgary, Calgary, AB T2N 4N1, Canada

**Keywords:** physical activity, behaviour change, health communication, older adults

## Abstract

We compared perceptions of enablers, barriers, and motivators to greater physical activity by older adults in two respondent groups: individuals 55+ years of age participating in a research exercise program and exercise professionals who plan and deliver programming to older adults. We developed and administered a questionnaire on potential factors influencing physical activity participation among older adults. Questionnaire items were transformed into scales and analyzed using independent sample Mann–Whitney *U* tests and principal component analyses (PCA). Statistically significant differences emerged between the respondent groups. Compared to older adults, exercise professionals rated the influence of physical capabilities (*p* < 0.001), social (*p* < 0.001) and physical opportunities (*p* < 0.001), and reflective motivations on barriers to physical activity (*p* < 0.001) higher. Older adults rated reflecting on the consequences of physical inactivity (*p* < 0.05) higher. Respondent groups differed in their perspectives regarding the relative influence of enablers, barriers, and motivators to physical activity participation, and these differences may inform physical activity messaging for older adults.

## 1. Introduction

Being more physically active plays a vital role in maintaining health and function in older age [1,2]. There is strong evidence that greater physical activity and exercise are associated with a higher likelihood of maintaining intact cognition as we age [3,4,5,6,7], higher health-related quality of life [8], and less functional loss and frailty [9,10,11]. However, many older adults do not meet the recommended physical activity levels found in guidelines for individuals 65 years of age and older [12,13].

The physical activity rates found at older ages are, in general, below what is recommended by guidelines [12,14,15,16]. Public health messaging, encouragement by physicians and other healthcare providers, and supportive communities and built environments are just a few of the strategies used to increase physical activity participation [17,18,19]. Ensuring that the messaging regarding physical activity better resonates with the target audience is another strategy. A greater understanding of the perceived benefits and challenges to participating in physical activity may allow for the creation of targeted messaging that will increase the likelihood of those messages sticking, with a resulting increase in physical activity rates and the related positive health benefits.

Resulting from a synthesis of existing behaviour change frameworks, Michie et al. [20] proposed the Behaviour Change Wheel. Central to the wheel is a hub—the COM-B system, a framework for understanding behaviour. The framework proposes that behaviour (B) requires three essential conditions: capability (C), opportunity (O), and motivation (M). Each of these three conditions has two subcomponents (i.e., physical and psychological capability, physical and social opportunity, and reflective and automatic motivational processes, respectively). The COM-B framework may help identify areas requiring attention to support or promote physical activity behaviours. That is, the use of this system as a foundation for further exploration may suggest targets to influence motivation to increase a desired behaviour.

We used this work [20] as a starting place in our inquiry about beliefs regarding physical activity participation in older adulthood. To improve messaging to promote participation, it would be useful to understand existing perceptions regarding the benefits, challenges, facilitators, and barriers to such behaviours. Constructs aligned with the COM-B framework may be effective as messages for physical activity behaviours, either to promote behaviour change or as messaging to reinforce existing behaviours. However, it is not known how the constructs resonate with older adults, nor how well the physical activity beliefs of older adults and exercise professionals align or contrast. Therefore, to inform these questions, the aim of this quantitative descriptive study was to compare perceptions about physical activity among older adults held by two respondent groups: individuals 55 years of age and older and the exercise professionals who plan and deliver programming to them.

## 2. Materials and Methods

We undertook the development and administration of a questionnaire to compare the factors influencing physical activity participation among older adults as perceived by a convenience sample of exercise professionals and older adult exercise participants in a research study. All participants provided informed consent prior to participating. This study received approval from the Conjoint Health Research Ethics Board at Unversity of Calgary (REB# 14-2284).

### 2.1. Questionnaire Design

Questionnaire development began in advance of a public community engagement event addressing the topic of active living and brain health. Nineteen predefined questions were devised by the study team to serve as discussion prompts about physical activity and exercise participation within a planned session of the event. Questions aligned with the domains of (a) capability, (b) opportunity, and (c) motivation, following the COM-B framework of the Behaviour Change Wheel [20].

Just over 200 registrants attended the community engagement event. Within the event, attendees were invited to participate in an interactive session to identify and discuss potential barriers and motivators to achieving regular physical activity. Discussions were held in a World Café format for hosting large group dialogue [21]. The World Café approach can be applied to group settings in which community participation is required [22]. Attendees began at one of nineteen tables and participated in a discussion on one of the predefined questions for 20 min. Two researchers were present at each table, one to moderate the discussion and the other to serve as a scribe to capture the discussion. At the end of the first round, the moderator and scribe remained at the table and attendees moved to different tables to form new groups discussing a different question. A final third round of discussions also following this format was then held. At the end of the event, the hosts and scribes gathered and shared collective discoveries.

Scribed content from World Café discussions was summarized and categorized into statements, which were then incorporated into a questionnaire aimed at identifying the barriers and motivators to achieving regular physical activity. The questionnaire was reviewed by the team and a non-involved older adult for clarity of wording and readability prior to finalization. A total of 86 items in 19 sub-sections were included in the final version.

### 2.2. Questionnaire Administration

The theoretically informed questionnaire designed above was distributed to two groups using non-probability sampling. One was an older (55 years of age and older) adult group participating in a group exercise research trial [23,24]. The second group consisted of exercise professionals involved in the planning and delivery of exercise programming to older adults (e.g., exercise physiologists, kinesiologists, and recreation therapists). The convenience sample of older adults was asked to voluntarily complete the questionnaire online using SurveyMonkey online survey software, while the convenience sample of exercise professionals was asked to voluntarily complete a paper copy of the questionnaire at a pre-conference symposium that was part of a provincial exercise, health, and fitness-focused conference they had registered to attend (thus, they were a pre-determined sample based on their registration). Older adults were asked to respond to the statements by thinking of themselves and their personal experiences. Exercise professionals were asked to respond according to the extent to which they believed each statement reflected the thoughts of older adults. In both formats, participants were asked to rate their level of agreement with each statement presented as a 5-point Likert item (strongly disagree = 1; undecided = 3; strongly agree = 5).

### 2.3. Data Analysis

The median and interquartile range were calculated to identify the central tendencies for each item. We used the intraclass correlation coefficient (ICC) to assess the unidimensionality of the individual questionnaire items (*n* = 86). Items with a Cronbach’s alpha of ≥0.7 were retained. The remaining items were transformed into a Likert scale using the mean of the included Likert items.

Independent sample Mann–Whitney *U* tests for data in a non-normal distribution were used to determine if there were any statistically significant differences in Likert scale responses between the two respondent groups. Bonferroni’s correction was applied to conservatively address the multiple comparisons [25]. Finally, we employed a principal component analysis with varimax rotation to explore the reduction of the questionnaire scales into underlying principal components. In recognition of our small sample size, we employed a subject-to-variable ratio of 10:1. A parallel analysis technique was used to determine the number of components to retain [26]. All data were analyzed using SPSS Statistics for Windows, version 19 (IBM Corp., Armonk, NY, USA).

## 3. Results

In total, data from 78 individuals were obtained (exercise professionals *n* = 28, 36%; older adult exercise participants *n* = 50, 64%). The mean age (*SD*) of the exercise professionals completing the questionnaire was 45.8 years (11.1 years), and their mean years of experience ranged from <1 year to 37 years (*M* = 15.9 years, *SD* = 10.6 years). No information was collected on non-respondents. Sociodemographic characteristics of the older adult respondent group were not collected, although it was known that all respondents and non-respondents were 55 years of age or older [23].

Median and interquartile ranges for the individual questionnaire Likert items can be found in Table 1. The data reduction approaches that were used led to eight Likert scales (Table 2). The scales addressed the psychological capability (*n* = 3), physical capability (*n* = 1), social opportunity (*n* = 1), physical opportunity (*n* = 1), and reflective motivation (*n* = 2) domains of the COM-B framework [20,27]. No data were retained for the automatic motivation domain.

**Figure 1:**
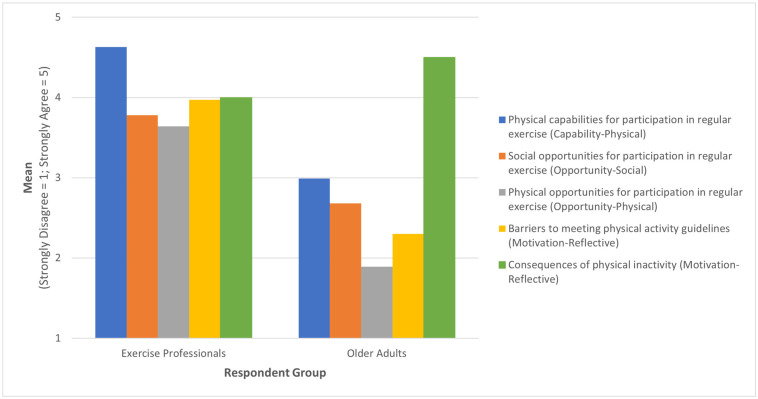
Statistically significant differences in mean Likert scale ratings of perspectives of older adults’ physical activity participation by two respondent groups.

Statistically significant differences in Likert scale responses were found between the respondent groups (Figure 1). Compared to older adults, exercise professionals gave higher scores on four of the five scales. Exercise professionals more strongly agreed with the influence of physical capabilities on participation in regular exercise than older adult respondents (*U* = 1299, *p* < 0.001). They also indicated stronger agreement on the influence of both social and physical opportunities for participation in regular exercise than older adults (*U* = 1214.5, *p* < 0.001, and *U* = 1254.5, *p* < 0.001, respectively). Finally, exercise professionals reported stronger agreement on the importance of barriers to meeting physical activity guidelines within the reflective motivational domain (*U* = 1253.5, *p* < 0.001). Regarding the consequences of physical inactivity, the other scale within the reflective motivational domain, older adults reported stronger agreement about the association between deterioration and not being physically active than the exercise professionals (*U* = 366.5, *p* < 0.05).

A principal component analysis was used to explore the Likert scale data. The Kaiser–Meyer–Olkin measure of sampling adequacy was 0.77 (above the commonly recommended value of 0.6), and Bartlett’s test of sphericity was significant (χ^2^ (28) = 307.5 *p* < 0.001), indicating that the data were appropriate for analysis. The principal component analysis using varimax rotation identified two components derived from the eight variables that explained 68.7% of the variance. Component 1 included the physical capability, social and physical opportunity, and reflective motivation domains. It represented challenges to physical activity participation (Table 3). Component 2 included scales in the psychological capability and reflective motivation domains and can be conceptualized as representing the importance of physical activity (Table 3).

## 4. Discussion

We developed a theoretically informed questionnaire and administered it in two respondent groups. This study demonstrated the differences in physical activity perceptions between older adult exercisers and exercise professionals who plan and deliver programming to this population. Our novel approach to informing health communication messaging was grounded in both theory and stakeholder insights of those who provide and participate in physical activity programming.

We found statistically significant differences between exercise professionals’ and older adults’ responses in a few noteworthy scales. First, our sample of exercise professionals rated the influence of the physical capabilities domain on participation in regular physical activity higher than the older adult group. The physical capabilities domain included factors, such as fatigue, and musculoskeletal issues like joint pain. This finding suggests that exercise professionals perceive the physical capabilities of older adults as influencing physical activity behaviour more than older adults themselves. In practice, this perception may result in exercise professionals inadvertently assuming modifications are necessary to address fatigue or joint pain when that may not necessarily be the case. Older adults are a heterogeneous group [28], and providers should avoid generalizations about capabilities in favour of individually tailored support [29,30], as would be appropriate in other age categories across the life course.

Another area of difference was in the social and physical opportunity domains. Exercise professionals rated these scales as more strongly influential to participation in regular physical activity than older adults. These scales included factors such as other commitments hindering physical activity, supportive social relationships, or others with whom one can be physically active. Exercise professionals also rated the barriers associated with meeting guidelines more highly than older adults. Within the reflective motivational domain, time constraints, seasonal restrictions and weather conditions, and safety (both general and in relation to existing medical conditions) are considered. Again, the higher ratings by exercise professionals suggest a mismatch in perspectives that may influence the support and encouragement of physical activity participation during interactions with older adults. These factors can influence older adults’ physical activity participation [31], but exercise professionals rated their influence even more strongly than older adult respondents. Therefore, prompting for activity buddies or planning for alternate activities in poor weather may not support the uptake or continuation of physical activity in the manner intended if these same factors are not viewed by older adults to be as influential as exercise professionals perceive them to be.

Although exercise professionals reported stronger agreement on the abovementioned scales than older adult respondents, an interesting reversal was found on one scale. Exercise professionals rated less strongly the influence of the protective effects of physical activity in older adulthood. Exercise professionals scored significantly lower than older adult respondents regarding the influence of physical and mental health deteriorations that are associated with not being physically active, highlighting a potentially important difference in perspectives. This suggests that a strong driver of physical activity behaviours among older adult respondents was the perceived functional and cognitive declines brought on by inactivity. Maintaining physical and cognitive function, as part of healthy aging, may be a substantial motivating reason for engaging in physical activity behaviours. Our finding parallels those of others [32,33,34], who reported that the maintenance of health was a significant motivator of older adults’ physical activity participation. It is possible that this reflective motivation may offer a strong base on which effective messaging for physical activity promotion could be built.

A two-component structure for the eight items was shown in the principal component analysis and represented challenges to physical activity participation and the importance of physical activity, accounting for the majority of meaningful variance. The Likert scales that loaded onto Component 1, challenges to physical activity participation, included the four scales that were rated statistically significantly higher by exercise professionals than older adult exercisers. This suggests that the role of the associated COM-B components [20,27], physical and social opportunities, physical capabilities, and reflective motivation, on physical activity participation was not perceived to hold equal relevance by respondents of the two groups. In practice, this may suggest that exercise professionals who try to promote physical activity participation by addressing these domains may be missing the mark with an audience that does not perceive these issues as influential on their behaviour in the first place. Although individual motivations for participating in physical activity vary [34], Component 2 of our findings would suggest that the importance of physical activity for physical and brain health may be a strong basis for messaging about physical activity for older adults.

We grounded the questionnaire development in the behaviour change theory using the COM-B framework and Behaviour Change Wheel [20]. Our findings suggest possible constructs for intervention to promote physical activity behaviours among older adults. One potential is to better target messaging [35] from exercise professionals and, potentially, other health promotion avenues to the domains that influence older adults’ decision-making regarding physical activity behaviours. For example, strategically incorporating messaging regarding the substantial benefits of activity and exercise for physical and cognitive health may more strongly resonate with older adults. This finding parallels the work of Preissner and colleagues [36], whose findings also suggest that health providers may find utility in promoting the benefits of physical activity to support participation. Another strategy may be to align strategies or interventions from the Behaviour Change Wheel with the Likert scales that produce statistically different response patterns between our two groups. As an example, the consequences of physical inactivity on the reflective motivation domain that were scored more highly by older adults than exercise professionals may present an opportunity to further test interventions to support behavioural uptake, such as education, persuasion, and incentivization [20,37,38]. Beyond the implementation of our findings into practice, this study has also suggested potential opportunities for future research. Further investigation may be useful into how individual characteristics, such as the chronic disease status of older adult exercisers or the years of experience and education of exercise professionals, might influence perspectives and opinions regarding physical activity participation.

We recognize that there are limitations to this study. First, our older adult respondent group was recruited from an existing exercise trial. The perspectives held by those already participating in regular exercise may not be generalizable to all older adults, as the facilitators and barriers to physical activity participation may be different for those not yet engaging in such behaviours. Additional research would be needed to compare and contrast the findings of this study with the perspectives of non-exercisers. Second, we acknowledge that this work was an exploratory comparison of perspectives held by two small subgroups. We recognize that further validation of our findings is necessary. However, this study offers preliminary findings worthy of further consideration regarding constructs relevant to the motivations for and decision-making regarding physical activity behaviours. It illuminated the perspectives of older adult exercisers on the enablers and barriers to their participation in physical activity, as well as the related perspectives of exercise professionals that may come to bear within their practice.

## 5. Conclusions

This exploratory study compared the perspectives regarding physical activity held by two respondent groups: older adults and the exercise professionals who plan and deliver programming to them. We found differences in group perspectives regarding the relative influence of enablers, barriers, and motivators relating to physical activity participation. These differences may be informative in understanding behaviours and targeting messaging or interventions to encourage the uptake of physical activity behaviours or the maintenance of those behaviours once initiated.

## Figures and Tables

**Table 1:** Comparison of median and interquartile range corresponding to each Likert item, as reported by the exercise professionals and older adult respondent groups.

Likert Item ∗	Exercise Professionals			Older Adults		
	(*n* = 28)			(*n* = 50)		
	Median	IQR	Valid *n*	Median	IQR	Valid *n*
**CAPABILITY—Psychological**						
**2. Is physical activity important for brain health? Why or why not? What do you know about the role of physical activity in maintaining brain health?**						
Physical activity is important for brain health because it increases blood flow to the brain.	4	2	28	5	1	50
Physical activity is important for brain health because it improves sleep.	4	2	28	4	1	50
Physical activity helps maintain brain health.	4	2	28	5	1	50
Physical activity will improve my memory.	4	2	28	4	2	50
Improved cardiovascular health will reduce the risk of stroke.	4.5	1	28	5	1	50
**3. Is there a way that physical activity can be promoted?**						
Community newsletters are a great way to promote physical activity.	4	1	28	3	1	50
Physical activity can be promoted with changes in by-laws.	3	1	27	3	1	49
Physical activity can be promoted by making communities ‘walkable’.	4	1	27	4	1	49
Community centers should start local programs to promote physical activity.	4	1	28	4	1	49
Educating the public on the benefits of physical activity will promote physical activity.	4	1	28	4	1	49
Friends/family can promote physical activity.	4	2	27	5	1	49
**4. Why is physical activity important to you?**						
It is important for me to participate in physical activity to be accountable for my health.	4	0	28	5	1	50
Physical activity is an important part of my social life.	4	1	28	4	2	50
Physical activity improves my mood.	4	1	28	5	3	50
**5. What goes into your/people’s decision-making when taking part in regular physical activity?**						
The most important factor to consider when taking part in regular physical activity is time commitment.	4	3	28	4	2	50
Cost and location are important factors to consider when deciding to take part in regular physical activity.	5	1	28	4	2	50
I think about my physical limitations prior to taking part in regular physical activity.	5	1	28	4	2	50
Employer flexibility is important when deciding whether or not to engage in regular physical activity.	4	2	26	4	1	46
**CAPABILITY—Physical**						
**6. How are you/others you know physically hindered from participating in regular physical activity?**						
I have joint pain/an injury that hinders my participation in regular physical activity.	5	1	28	3	2	49
Icy sidewalks make it challenging to be physically active in winter.	5	1	28	4	2	49
I am too tired to participate in regular physical activity.	5	1	28	2	2	49
**7. What types of skills do you feel would help you incorporate regular physical activity into daily life? What knowledge/skills are necessary for older adult’s participation in regular physical activity?**						
I find participating in exercise difficult because I do not know how to use the equipment.	4	1	28	2	1	48
Knowledge of my physical limits is necessary in order to participate in regular physical activity.	4	0	28	4	1	48
Knowledge of the benefits of physical activity is necessary in order to participate in regular physical activity.	4	1	28	4	2	48
Knowing where to find information on available programs/activities is important when deciding to participate in regular physical activity.	4	1	28	4	1	48
**OPPORTUNITY—Social**						
**8. What about your/one’s social situation may help or hinder you/them to be physically active? Is there anyone in your life that hinders you from being physically active?**						
Self-motivation is not enough to encourage participation in physical activity; I need motivation from others.	4	1	28	2	2	48
Family and friends can impact my motivation to be active (positively or negatively).	4	1	28	4	2	48
I am too busy with work/other commitments to be physically active.	4	1	28	2	1	48
The only person that hinders me from maintaining physical activity is myself.	3	2	28	4	1	47
Team sports motivate me to keep active.	3	1	28	2	2	47
It is difficult to meet people when retired; therefore, I find it difficult to find a group to be physically active with.	4	1	28	2	1	47
**9. Do you have friends/family that you can be physically active with? Is there someone to act as a guide or role model (someone to show you what/how to perform the activity)?**						
I can be physically active with my spouse/friends.	4	1	28	4	1	46
I do not have friends or family who I can be physically active with.	4	1	28	2	1	47
**10. What type of environment do you prefer to be physically active in (formal—at the gym, with others, alone, group exercise, outside, etc.)?**						
I prefer to be physically active in a group.	4	1	28	4	1	46
I prefer to be physically active outdoors.	4	2	28	4	1	47
I prefer to be physically alone.	3	2	28	2	1	46
I prefer to be physically active in a gym/fitness center.	3	2	28	3	2	47
**11. What past experiences with physical activity may help or hinder adherence to exercise recommendations?**						
I have injuries that hinder my adherence to exercise recommendations.	5	1	28	2	3	47
My neighbourhood is restrictive (e.g., no sidewalks), so I find it difficult to adhere to physical activity recommendations.	4	0	28	2	1	47
Having different physical activity options helps me in meeting exercise recommendations.	4	0	28	4	1	47
Having support from my friends, family, and/or employer helps me in meeting exercise recommendations.	4	1	28	4	0	46
Having many physical activity options is helpful in adhering to exercise recommendations.	4	1	28	4	1	47
**OPPORTUNITY—Physical**						
**12. What about living in Calgary/Canada promotes or inhibits you/people from being physically active?**						
Fear of falling (icy sidewalks) deter people from being physically active in winter.	5	1	26	4	0	47
More education on the importance of physical activity would promote physical activity in Calgary.	4	1	26	4	1	47
High costs associated with physical exercise (e.g., gym fees and transportation) limit my participation in physical activity.	4	1	26	2	2	47
Making communities ‘walkable’ would promote physical activity in Calgary.	4	0	26	4	1	47
Calgary is close to National Parks, and this promotes physical activity.	3	2	25	4	1	47
**13. Do you have any commitments that may hinder you from being physically active?**						
I am a caregiver and therefore cannot participate in regular physical activity.	4	1	28	2	1	44
I have work/volunteering commitments that hinder my participation in regular physical activity.	4	1	28	2	1	47
I have no time commitments; I just have not found a physical activity I enjoy.	4	1	28	2	2	47
**14. Are there sufficient facilities where you feel comfortable being physically active? How accessible are facilities for you?**						
I am uncomfortable being physically active in a gym.	4	0	28	2	2	47
Calgary offers many facilities to promote physical activity, but the associated costs are too high.	4	1	23	4	2	47
There is a lack of senior-friendly advertising regarding facilities/activities that Calgary has to offer, so I am not aware of what is available.	4	1	23	4	2	45
There are not enough facilities to be physically active in Calgary—every community should have one.	3	2	24	3	2	46
**MOTIVATION—Reflective**						
**15. What do you think may happen if you are not physically active?**						
My physical health will deteriorate if I am not physically active.	4	1	27	5	1	47
My mental health will deteriorate if I am not physically active.	4	1	27	5	1	47
**16. Do you think it would be difficult to meet the physical activity guidelines? Why/why not? What problems have you encountered that prevent you from being physically active?**						
It is difficult to meet physical activity guidelines in the winter.	5	1	27	2	2	46
Time constraints make it difficult to participate in physical activity and meet recommended guidelines.	4	1	27	2	1	47
Other activities are more important to me than physical activity.	4	1	27	2	1	46
Weather conditions create fear for me to perform outdoor activities.	5	1	27	2	2	47
I am not sure what is safe to participate in with my medical conditions.	5	1	27	2	2	46
Self-care is not a focus for my generation.	3	2	27	2	1	45
I do not see a tangible outcome from physical activity; therefore, I do not participate.	3	2	27	2	1	47
I find it difficult to find activities that are pain-free with my medical conditions.	4	1	27	2	2	47
I lack peer support to continue with physical activity.	4	1	27	2	2	46
**17. What are some motivators to be physically active? Would having incentives along the way help you participate in daily physical activity? What types of incentives would you look forward to?**						
I am physically active because I have made a commitment to someone/a group.	4	1	26	2	2	47
I enjoy the social aspect of physical activity.	5	1	26	4	1	47
Technology helps keep me motivated to be physically active.	3	1	26	3	2	47
Meeting new people is a motivator for being physically active.	4	0	26	4	1	47
Controlling chronic conditions is a motivator to be physically active.	4	0	26	4	0	47
Incentives, such as tax breaks or free gym passes, would motivate me to be physically active.	4	1	26	4	2	45
**18. What helps you/people follow through with the intention of being physically active on a regular basis?**						
Scheduling exercise helps me follow through with the intention of being physically active.	4	0	26	4	1	47
Making small lifestyle changes (e.g., public transport, taking the stairs) helps me follow through with the intention of being physically active.	4	0	26	4	0	46
Receiving positive feedback from friends/family helps me follow through with the intention of being physically active.	4	0	26	4	0	46
Making commitments to others helps me follow through with the intention of being physically active.	4	0	26	4	2	47
**MOTIVATION—Automatic**						
**19. What are your feelings about exercising three times a week? How do you feel when you exercise/when you do not exercise? How do these feelings impact whether you exercise or not?**						
I know I have to exercise 3 times a week but choose not to or find it difficult to do so.	4	1	27	2	3	47
It is easier to exercise 3 times a week when you enjoy the activity.	4	1	27	4	1	47
It is easier to exercise 3 times a week when I have an end goal.	3	1	26	4	1	47
Time and money constraints make it difficult to exercise 3 times a week.	4	0	26	2	2	47
I do not enjoy being active, so I do not exercise 3 times a week.	4	1	27	2	1	47
I need to find an activity that I enjoy in order to consistently exercise.	4	1	27	4	2	47
I have more energy when I exercise.	4	0	27	4	1	47
I am grumpy when I do not exercise.	4	1	26	3	2	47
**20. Does physical activity fit into how you see your role in life (i.e., a mother, father, grandparent, working professional, etc.)? How can physical activity be incorporated into perceived role(s)?**						
I am a grandparent; therefore, I am more physically active when I spend time outdoors with my grandchildren.	4	0	27	3	2	37
I have hobbies that involve physical activity.	3	1	27	4	2	46
My job/volunteer position involves physical activity.	4	1	26	3	2	42

* Note: Bold text indicates the 19 predefined discussion prompts of the World Café conversations. Normal text indicates the statements generated by that discussion administered as a questionnaire to both respondent groups.

**Table 2:** Comparison of median and interquartile range corresponding to each Likert Scale, by respondent group.

Likert Scale		Exercise Professionals			Older Adults		
		(*n* = 28)			(*n* = 50)		
		Median	IQR	Valid *n*	Median	IQR	Valid *n*
CAPABILITY—Psychological							
	S2. Knowledge of physical activity for maintenance of brain health	4.1	2	28	4.6	1	50
	S3. Promoting physical activity	3.9	1	28	3.8	1	50
	S4. Importance of physical activity	4.0	1	28	4.5	1	50
CAPABILITY—Physical							
	S6. Physical capabilities for participation in regular exercise **	4.7	1	28	3.0	1	50
OPPORTUNITY—Social							
	S8. Social opportunities for participation in regular exercise **	3.8	0	28	2.7	1	48
OPPORTUNITY—Physical							
	S13. Physical opportunities for participation in regular exercise **	3.7	1	28	2.0	1	47
MOTIVATION—Reflective							
	S15. Consequences of physical inactivity *	4.0	1	27	5.0	1	47
	S16. Barriers to meeting physical activity guidelines **	3.9	0	27	2.3	1	47

Notes: * indicates statistical significance at the *p* < 0.05 level. ** indicates statistical significance at the *p* < 0.001 level.

**Table 3:** Components 1 and 2 from principal component analysis exploring Likert scale data from all participants.

Likert Scale	Domain	Component 1: Challenges to Physical Activity Participation	Component 2: Importance of Physical Activity
Barriers to meeting physical activity guidelines	Reflective Motivation	0.925	−0.197
Commitments hindering physical activity	Physical Opportunity	0.884	−0.106
Physical barriers to regular physical activity	Physical Capability	0.874	−0.156
Social barriers to physical activity	Social Opportunity	0.787	−0.015
Physical activity promotion	Psychological Capability	0.196	0.844
Personal importance of physical activity	Psychological Capability	−0.322	0.763
Importance of physical activity for brain health	Psychological Capability	−0.125	0.728
Consequences of physical inactivity	Reflective Motivation	−0.413	0.503

## Data Availability

Study data supporting the findings of this research are available upon reasonable request from the corresponding author in compliance with Research Ethics Board approval.
